# Measuring the mass, volume, and density of microgram-sized objects in fluid

**DOI:** 10.1371/journal.pone.0174068

**Published:** 2017-04-05

**Authors:** Shirin Mesbah Oskui, Heran C. Bhakta, Graciel Diamante, Huinan Liu, Daniel Schlenk, William H. Grover

**Affiliations:** 1 Department of Bioengineering, University of California, Riverside, Riverside, CA, United States of America, 92521; 2 Department of Environmental Sciences, University of California, Riverside, Riverside, CA, United States of America, 92521; Texas A&M University College Station, UNITED STATES

## Abstract

Measurements of an object’s fundamental physical properties like mass, volume, and density can offer valuable insights into the composition and state of the object. However, many important biological samples reside in a liquid environment where it is difficult to accurately measure their physical properties. We show that by using a simple piece of glass tubing and some inexpensive off-the-shelf electronics, we can create a sensor that can measure the mass, volume, and density of microgram-sized biological samples in their native liquid environment. As a proof-of-concept, we use this sensor to measure mass changes in zebrafish embryos reacting to toxicant exposure, density changes in seeds undergoing rehydration and germination, and degradation rates of biomaterials used in medical implants. Since all objects have these physical properties, this sensor has immediate applications in a wide variety of different fields including developmental biology, toxicology, materials science, plant science, and many others.

## Introduction

One of the most fundamental and important descriptions of an object is its *weight*. Unlike other measurements of an object’s size, weight is not influenced by the shape of the object. Consequently, for at least 4000 years weight has been the basis for trade of goods and materials. And for living organisms, weight measurements provide valuable insights into an organism’s biological state and health. We measure the weight of our children from birth and plot these measurements over time on growth charts; any deviation from expected growth can indicate a health problem. People, birds [1], fish [2], and plants [3] are just a few of the organisms that are weighed to study their health and growth. But while children, animals, and plants can be weighed on a scale, some biological samples are more challenging to weigh. Samples like single cells, microorganisms, and embryos are too small to weigh on conventional laboratory balances, and these samples often live in a liquid environment that is incompatible with conventional scales.

Weighing the smallest living samples in their native liquid environments became possible with the advent of the suspended microchannel resonator or SMR [4]. The SMR consists of a microfluidic channel embedded inside a silicon cantilever. The cantilever is vibrated at its resonance frequency, which is a function of the overall mass of the cantilever. When a cell flows through the embedded microfluidic channel, the cell’s mass momentarily increases the cantilever’s mass. This momentarily decreases the resonance frequency of the cantilever by an amount proportional to the weight of the cell. By weighing cells in this manner, the SMR can be used to distinguish healthy and diseased cells, monitor cell growth, and observe the effects of drugs on cells [5–7].

However, there are many important samples that are too large to be weighed in the microfluidic SMR and too small to be weighed on a conventional balance. For example, fish embryos are used in a variety of developmental biology and toxicology applications [8]. If the weight of an embryo could be monitored as it grows or reacts to stimuli, these measurements could offer new insights into developmental biology and toxicology. However, millimeter-sized zebrafish embryos are far too large for the micron-scale channels in the SMR, and the aquatic environment of the embryos complicates weighing them using a traditional balance. In another example, materials with controlled degradation rates are finding important applications in medical implants, where they remain in the body for a predetermined period of time and then effectively disappear [9]. But accurately predicting the degradation rates of these materials in the fluidic environment of the body is challenging. If the weight of a biomaterial sample could be monitored as it degrades in a realistic artificial bodily fluid, these measurements would provide an unparalleled insight into the degradation of the material within the body. However, with their liquid environments and small size, these biomaterial samples are difficult to weigh using existing techniques, and manually monitoring the weight of these samples over weeks or months of degradation is a laborious and time-consuming process.

**Fig 1:**
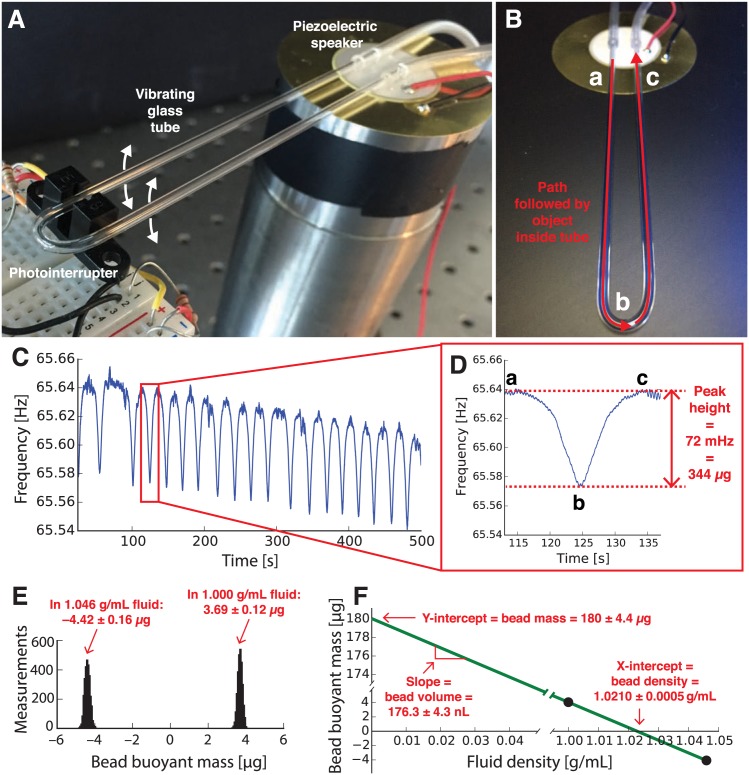
Using a vibrating glass tube to weigh objects in fluid. **(A)** The tube is bent into a “U” shape and mounted on an inexpensive piezoelectric speaker. The resonance frequency of the tube is detected by a photointerrupter, amplified, and fed into the speaker; the resulting feedback circuit keeps the glass tube vibrating at its resonance frequency. A peristaltic pump and computer (not shown) are used to flow samples through the tube and record the resonance frequency of the tube. **(B)** Detail of the glass tube shows the path followed by a sample inside the tube (red line) as it flows into the sensor (“a”), passes the tip of the sensor (“b”), and exits the sensor (“c”). **(C)** Resonance frequency of the vibrating tube vs. time as a 770 *μ*m diameter glass bead is passed back and forth through the tube eighteen times. Each passage of the bead through the tube results in a momentary decrease in the tube’s resonance frequency; this is recorded as a downward peak in the resonance frequency **(D)**. Each point on the peak (baseline “a,” tip “b,” and baseline “c”) corresponds with the bead’s location in (B) above. The height of this peak (72 millihertz) is used to determine the buoyant mass of the bead (344 micrograms). **(E)** Histograms showing the buoyant mass of another bead weighed thousands of times in two different fluid densities. In deionized water (density 1.000 g/mL) the bead has an average buoyant mass of 3.69 ±0.12 *μ*g, and in a sodium chloride solution (density 1.046 g/mL) the bead has an average buoyant mass of −4.42 ±0.16 *μ*g. The widths of these distributions—120 and 160 nanograms—provide an estimate of the resolution of our mass measurements. **(F)** By plotting the average buoyant mass of the bead vs. the density of the fluid in which the bead was measured from (E) and drawing a line between the two points, we can determine the absolute mass (180 ±4.4 *μ*g), volume (176.3 ±4.3 nL), and density (1.0210 ±0.0005 g/mL) of the bead from the y-intercept, slope, and x-intercept, respectively. The measured bead density is in good agreement with the manufacturer-provided value of 1.023 g/mL, and the bead diameter calculated from the measured volume of the bead (700 *μ*m) is very close to the measured diameter of the bead obtained using a digital caliper (710 *μ*m).

In this work, we demonstrate a simple and inexpensive sensor capable of weighing microgram-sized objects in fluid. Like the SMR, this sensor uses a change in resonance frequency to weigh an object in fluid with high precision. But unlike the SMR, this sensor can weigh samples with a large range of sizes and is extremely simple to fabricate. Our sensor consists of a short length of glass tubing bent into a “U” shape and attached to an inexpensive speaker that vibrates the glass tubing at its resonance frequency. The resulting sensor shown in Fig 1 costs about US $12 in materials and can be made in under 10 minutes. Additionally, by weighing samples in fluids of different densities, we can also use our sensor to measure the volume and density of samples in fluid. In this proof-of-concept demonstration, we used vibrating glass tubes to monitor fish embryos reacting to toxicant exposure, plant seeds undergoing rehydration and sprouting, and biomaterials undergoing controlled degradation.

## Theory

In vibrating mass sensors, the resonance frequency of the vibrating sensor is inversely proportional to the effective mass of the sensor. For a vibrating sensor with a cantilever or “diving board” shape, the resonance frequency *f* of the sensor is

$$\begin{array}{c} f=\frac{1}{2\pi }\sqrt{\frac{k}{m_{s}}} \end{array}$$ (1)

where *m*_*s*_ is the effective mass of the sensor and *k* is the spring constant of the sensor [4]. If an additional mass is added to the sensor, the increase in *m*_*s*_ in Eq (1) causes a measurable decrease in the cantilever’s resonance frequency *f*. For vibrating mass sensors containing a fluid-filled channel (like the vibrating tubes presented here), the contents of the channel contribute to the sensor’s mass and therefore affect its resonance frequency. The resonance frequency of the sensor is inversely proportional to the density of the fluid filling the channel; this is the basis for the long-established technique of using vibrating tubes to measure fluid density [10], and when two immiscible fluids are used, vibrating capillaries can be used to measure the density and radius of droplets of fluid in the tube [11].

In this work, we show that any object (not just fluids) flowing through a vibrating tube can affect the resonance frequency of the tube. Since the object is flowing in fluid, the mass it contributes to the vibrating tube is actually the object’s *buoyant mass*, *m*_*bo*_, defined as

$$\begin{array}{c} m_{bo}=m_{o}(1-\frac{\rho_{f}}{\rho_{o}}) \end{array}$$ (2)

where *m*_*o*_ is the absolute (*in vacuo*) mass of the object, *ρ*_*o*_ is the density of the object, and *ρ*_*f*_ is the density of the fluid filling the channel. Stated in words, an object’s buoyant mass is equal to its real mass minus the mass of an equivalent-volume amount of fluid. If the object’s density is *greater* than the fluid’s density, then the object has a positive buoyant mass and its passage through the vibrating tube will be recorded as a momentary *decrease* in the tube’s resonance frequency (the downward peaks shown in Fig 1C and 1D). If the object’s density is *less* than the fluid’s density, then the object has a negative buoyant mass and its passage through the tube will result in a momentary *increase* in the tube’s resonance frequency (upward peaks). Finally, if the object’s density *equals* the fluid’s density, then the object will have zero buoyant mass and its passage through the tube will have no effect on the resonance frequency of the tube (although this situation can be easily avoided by changing the fluid density). Note that while the vibrating tube sensor is sensitive to an object’s *buoyant mass*, it is not affected by *buoyant forces* (that cause an object to sink or float relative to gravity) because the object being measured is confined to the tube and cannot sink or float vertically. Thus, the orientation of the vibrating tube with respect to gravity has no effect on its measurements.

In addition to buoyant mass, the vibrating tube sensor can measure other physical properties of an object. For example, by measuring the buoyant mass of an object in two fluids of different densities, we obtain two instances of Eq (2) that can be solved simultaneously to calculate the absolute (*in vacuo*) mass *m*_*o*_ of the object, the density *ρ*_*o*_ of the object, and the volume *V*_*o*_ of the object (from the definition of density, *ρ*_*o*_ = *m*_*o*_/*V*_*o*_) [7]. A graphical version of this calculation is shown in Fig 1E and 1F: by plotting two measurements of an object’s buoyant mass vs. the density of the fluid in which it was weighed and connecting the points with a line, the mass of the object is equal to the y-intercept of the line, the density of the object is equal to the x-intercept of the line, and the volume of the object is equal to the slope of the line.

## Materials and methods

### Sensor fabrication and oscillator circuit design

To fabricate vibrating glass tube sensors, glass tubing (1.50 mm inner diameter, 1.80 mm outer diameter; VitroCom, Mountain Lakes, NJ) was cut to length and bent into a “U” shape using a butane torch. The resulting sensors have a mass of around 1.4 g empty and 1.7 g when filled with water. Our technique is sensitive to the amount of change in the sensor’s mass when an object flows through the sensor, so it is generally advantageous to maximize this change by keeping the mass of the tube as small as possible.

The top of the “U”-shaped glass tube was attached to an inexpensive piezoelectric speaker (Jameco Electronics, Belmont, CA) using epoxy. The bottom or tip of the “U” was suspended in the slot of a photointerrupter (an inexpensive electronic component that uses a light-emitting diode and a light-sensing diode to detect the position of an object inside its slot). As the tube vibrates at its resonance frequency, it blocks the photointerrupter’s light beam once per oscillation; the resulting photointerrupter output is an AC signal with the same frequency as the tube’s vibration. When the output of the photointerrupter is amplified and fed into the piezoelectric speaker, the circuit will spontaneously and continuously vibrate the glass tube at its resonance frequency. Alternatively, for some experiments, vibrating glass tubes from commercial fluid density meters were used. Fluid density meters (*e.g.*, DMA 35, Anton Paar, Graz, Austria) were obtained second-hand and modified to isolate the vibrating glass tube and supporting electronics.

### Q-factor measurement

The quality factor or Q-factor of an oscillating system like our vibrating tube sensor is an important value for quantifying the sensor’s ability to measure mass. An oscillator with a higher Q-factor has a purer “tone,” and changes in this tone (when objects pass through the sensor) can be measured more precisely. We measured the Q-factor of our sensors using the ring-down method. A vibrating tube sensor with a known resonance frequency *f* was manually “pinged” by flicking the tube using a finger. Video of the vibrating tube was recorded from the side of the tube (to measure the decrease in vibrational amplitude over time) using an iPhone camera acquiring video at 240 frames per second. This video was imported into the software ImageJ [12] as an image stack and resliced vertically at the tip of the vibrating tube to create a bitmap that represents the tube’s vibrational amplitude on the Y axis and the frame number on the X axis (S5 Fig). We used ImageJ to measure the vibrational amplitude *A*_0_ of the tube at the start of the experiment (frame 0) and then located the frame number *n* at which the vibrational amplitude had decreased to a value of *A*_0_/*e* or about 37% of its initial amplitude. Dividing this number of frames *n* by the framerate of the camera yields is the exponential decay time *τ* of the sensor. Finally, the quality factor *Q* of the sensor was calculated using the relationship *Q* = *πfτ* [13].

### Data acquisition and analysis

To record the tube’s resonance frequency over time, we connected the output of the photointerruptor circuit to a counter input on a multifunction data acquisition device (National Instruments, Austin, TX) and a computer running a custom LabVIEW program, although we have also used simpler and cheaper hardware like the open-source Arduino microcontroller to measure the tube’s resonance frequency.

When a sample passes through the vibrating tube sensor, its buoyant mass is recorded as a brief peak in the plot of resonance frequency vs. time (*e.g.*, Fig 1C and 1D). To detect peaks in the data corresponding to object measurements, the raw frequency measurements were first filtered using a digital filter (either low-pass or Savitzky-Golay) and then subjected to a moving window average that identifies peaks based on their deviation from the baseline. Once a peak is located, the height of the peak can be measured and converted to a corresponding buoyant mass value using the sensor’s *point mass calibration* described below. Alternatively, a custom Python program can be used to fit the raw frequency measurements to an analytical equation of expected peak shape derived from Dohn *et al.* [14] The resulting buoyant mass measurements were recorded and processed using a moving window average filter with a window size of five data points to slightly reduce noise in the plots of buoyant mass vs. time. Additional details about signal processing (including sample frequency data before and after filtering) are provided in S6 Fig.

### Sensor calibration

Before use, each glass tube sensor was calibrated in two different ways. In the first calibration, the *fluid density calibration*, the tube was filled with different fluids of known density and the resonance frequency of the tube was recorded. Sodium chloride solutions with precisely known densities were prepared using our software tool *NaCl.py* [15]. By plotting resonance frequency vs. fluid density and fitting the plot to a line (S1 Fig), we obtain from the slope of that line a constant *c*_1_ that can be used to determine the density *ρ*_*f*_ of any fluid inside the tube as a function of the tube’s measured resonance frequency *f*:

$$\begin{array}{c} \rho_{f}=fc_{1} \end{array}$$ (3)

where *c*_1_ is the tube’s fluid density calibration constant in g mL^−1^ Hz^−1^. In the second calibration, the *point mass calibration*, the tube is filled with a fluid of known density *ρ*_*f*_. A microbead of known mass *m*_*o*_ and known density *ρ*_*o*_ is passed through the tube multiple times. The buoyant mass *m*_*bo*_ of the bead is calculated using Eq (2). As the microbead passes through the tube, the microbead’s mass momentarily changes the resonance frequency of the tube by an amount Δ*f* (the heights of the peaks in Fig 1C and 1D). We then solve for a constant *c*_2_ that can be used to determine the buoyant mass *m*_*bo*_ of any object inside the tube as a function of the measured change in resonance frequency Δ*f* as the object passes through the tube:

$$\begin{array}{c} m_{bo}=\Delta fc_{2} \end{array}$$ (4)

where *c*_2_ is the tube’s point mass calibration constant in g Hz^−1^.

Because of variation in the sizes of the vibrating glass tubes we used, the baseline resonance frequency *f* of the sensors used in this study ranged from below 100 Hz to around 500 Hz depending on the sensor size (larger sensors have a larger mass and therefore have lower resonance frequencies). Thus, each sensor has unique constants *c*_1_ and *c*_2_ which much be determined before use via the process described above. However, we have observed that a sensor’s calibration constants do not change significantly over long experiments (10 days of continuous operation), and some of our glass tube sensors have been in regular use for several years without a change in performance.

### Fluid control

To flow samples into and out of the vibrating glass tube sensors, the tubes were connected via flexible tubing to either a peristaltic pump or a servomotor-driven reservoir lifter. The reservoir lifter consists of two fluid reservoirs suspended from a rotary servomotor. By using the servo to raise one reservoir and lower the other, the resulting gravity-driven head pressure pumps fluid (and the sample) through the sensor. Reversing the positions of the reservoirs with the servo reverses the direction of fluid flow through the sensor. A custom LabVIEW program was used to continuously alternate the reservoir positions and measure the sample every few seconds.

### Measuring the buoyant mass of zebrafish embryos during toxicant exposure

**Fig 2:**
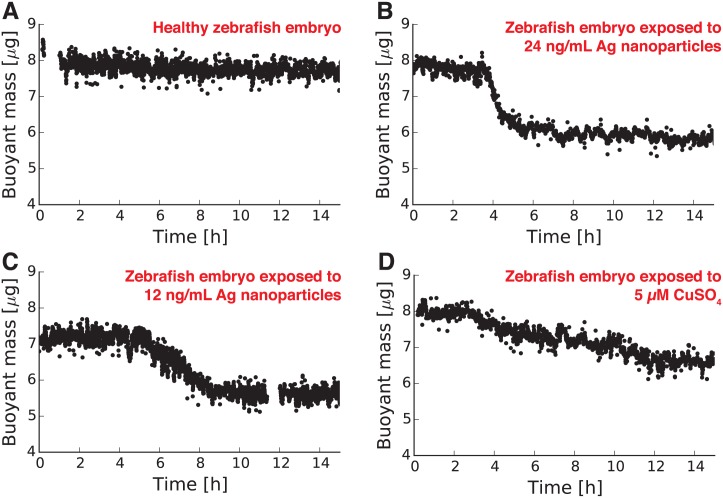
Monitoring the buoyant mass of single zebrafish embryos with a vibrating glass tube sensor. Most zebrafish embryos that were not exposed to toxicants have unchanging buoyant masses over the first 15 hours of their development **(A)**. However, embryos exposed to known toxicants (silver nanoparticles and copper sulfate) displayed clear decreases in their buoyant masses over the same time period **(B-D)**. Additional results from healthy and toxicant-exposed embryos are provided in *Supporting Information*.

This study was carried out in strict accordance with the recommendations in the Guide for the Care and Use of Laboratory Animals of the National Institutes of Health. Zebrafish (*D. rerio*) used in these experiments were purchased from Oregon State University and maintained following a protocol approved by the University of California, Riverside’s Animal Care and Use Committee (IACUC approval number 20130005). Wild-type AB-strain zebrafish were approximately 16 months old at the time of spawning and were kept in aerated aged tap water (dechlorinated) at a temperature of 27°C with a light/dark cycle of 14:10 hours. Males and females were kept separately and fed twice a day on *Artemia* sp. until the night before spawning, when they were transferred to breeding aquaria. Eggs were collected the next morning, examined, and separated based on the stage of the development. Measurements of embryo buoyant mass began at approximately 2 hours per fertilization. Using a peristaltic pump connected via tubing to the vibrating tube sensor, each embryo was passed back and forth through the sensor every 10 seconds for the duration of the experiment. The fluid surrounding the embryos was either water (Fig 2A and S3 Fig), a solution of silver nanoparticles (nanoComposix, San Diego, CA; B and C in Fig 2 and S4C–S4H Fig), or a solution of copper sulfate (Sigma Aldrich, St. Louis, MO; Fig 2D and S4A and S4B Fig). Embryo experiments were completed before hatching (and thus before the NIH Office of Laboratory Animal Welfare classifies the organisms as vertebrate animals). After each experiment, embryos were euthanized using either sodium hypochlorite solution or rapid freezing.

### Measuring the buoyant mass and density of seeds during imbibition and germination

**Fig 3:**
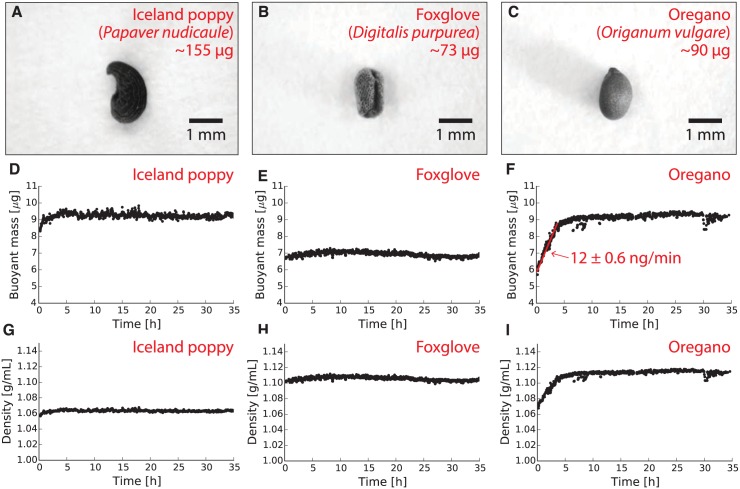
Using a vibrating tube mass sensor to measure the buoyant mass and density of three different types of seeds during imbibition and germination. Each individual seed was measured between 1,500 and 2,600 times over 35 hours. The largest seed, the Iceland poppy seed (**A**; *Papaver nudicaule*, ∼155 *μ*g mass), has the largest buoyant mass (9 *μ*g; **D**) but the smallest density (1.06 g/mL; **G**). The smallest seed, the foxglove (**B**; *Digitalis purpurea*, ∼73 *μ*g) had the smallest buoyant mass (7 *μ*g; **E**) but one of the largest densities (1.10 g/mL; **H**). Finally, the mid-sized oregano seed (**C**; *Origanum vulgare*, ∼90 *μ*g) has a buoyant mass that increases from 6 to 9 *μ*g at a rate of 12 ng/min during the first four hours of immersion (**F**); the density of the seed increases from 1.07 to 1.11 g/mL during the same time period (**I**).

Seeds of iceland poppy (*Papaver nudicaule*), oregano (*Origanum vulgare*), and foxglove (*Digitalis purpurea*) were obtained from Ferry-Morse (Norton, MA) and measured every 10 seconds in water until active germination (the emergence of the embryo) was observed using the servomotor reservoir lifter described above. The resulting buoyant mass measurements are shown in Fig 3D–3F. To estimate the density of these seeds during imbibition and germination, the average single-seed mass of each seed type was measured by weighing 30 seeds using a laboratory balance and dividing the total mass by 30. This estimate of single-seed mass was used along with the seed buoyant mass measurements to calculate the estimated density of each seed during imbibition and germination (Fig 3G–3I).

### Measuring the degradation of a biomaterial

**Fig 4:**
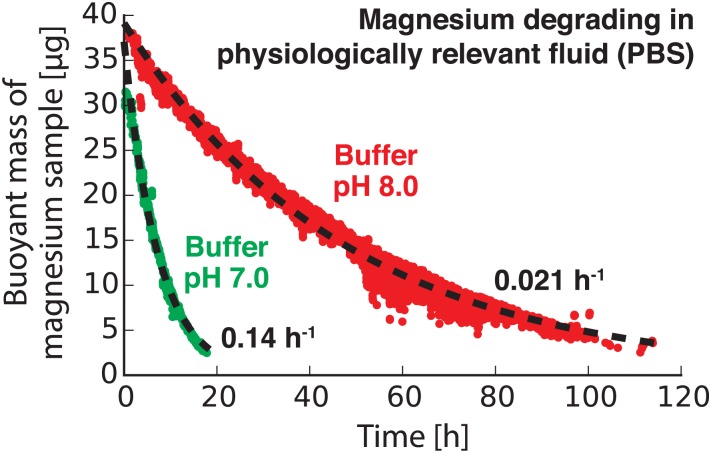
Using a resonating glass tube to measure the degradation rate of a sample biomaterial in different physiologically relevant fluids. Two millimiter-sized pieces of magnesium were measured in two different phosphate-buffered saline (PBS) solutions, one with a neutral pH (7.0; green) and one with a higher pH (8.0; red). The more acidic conditions at pH 7.0 caused the magnesium sample to degrade at a rate that is about six times faster than the sample in pH 8.0 buffer.

Magnesium ribbon with a thickness of 250 *μ*m (98% pure; MiniScience Inc., Clifton, NJ) was used as a model biomaterial in our degradation rate measurement studies. Roughly 1 mm sized pieces of magnesium were cut from the ribbon. The samples were polished before measurement using 600, 800, and 1200 grit silicon carbide abrasive papers to remove the native oxide layer. Each magnesium sample was then immersed in simulated bodily fluid (phosphate-buffered saline [PBS]) with adjusted pH (either 7 or 8) and passed back and forth through the vibrating tube sensor every 30 seconds until the piece had fully degraded. Flow through the sensor was controlled using the servomotor as described previously. The resulting buoyant mass measurements are shown in Fig 4.

## Results

Before using a vibrating tube to weigh a sample, the tube must first be calibrated. We calibrated the sensor shown in Fig 1A using a glass bead of known size and density in a fluid of known density (ethanol). Fig 1C shows eighteen downward peaks in the resonance frequency of the tube as the bead is pumped back and forth eighteen times through the tube. The height of each peak (72 millihertz; Fig 1D) is proportional to the buoyant mass of the bead (known to be 344 micrograms). From this relationship we obtain the calibration constant *c*_2_ for this tube sensor in micrograms per millihertz (4.8 *μ*g/mHz) that can subsequently be used to determine the buoyant mass of any object flowing through this tube. Our homemade vibrating tube mass sensors typically have quality factors around 500, which is comparable to that of tuning forks and high enough for precise measurement of the tube’s resonance frequency (S5 Fig).

In Fig 1C a slight downward drift is visible in the baseline resonance frequency of the vibrating tube over time; the frequency is decreasing at about 2 millihertz per minute. Slow baseline frequency drift like this is common in vibrating mass sensors and is mostly due to small fluctuations in the temperature of the sensor. However, the magnitude of this drift is small compared to the duration of a peak, which is why the baseline of an individual peak in Fig 1D is relatively flat. Additionally, since we use the *difference* in frequency between the baseline frequency and the tip of the peak (the peak height Δ*f*) to determine the buoyant mass of an object, this baseline drift does not affect the accuracy of our technique.

As an object passes through the vibrating tube sensor, the shape of each resulting peak in the tube’s resonance frequency (Fig 1D) is a function of the vibrational mode and amplitude of the tube. In this work, the tubes are vibrating at their primary vibrational mode, meaning that the amplitude of vibration is highest at the tip (the bottom of the glass “U”) and lowest at the base (the top of the “U”). When an object enters the tube (point “a” in Fig 1B), it is in a region with relatively low vibrational amplitude, so the object’s buoyant mass has a relatively small effect on the tube’s resonance frequency (point “a” in Fig 1D). However, as the object rounds the tip of the tube (point “b” in Fig 1B), the vibrational amplitude at the tip is the highest, so the object has a maximum effect on the tube’s resonance frequency here (point “b” in Fig 1D). As the particle leaves the tube (point “c” in Fig 1B) the tube’s vibrational amplitude in this region decreases again, so the resonance frequency of the tube returns to baseline (point “c” in Fig 1D). General mathematical expressions for predicting this peak shape for any vibrational mode were derived by Dohn *et al.* [14]

To determine the resolution of our technique, we used another glass tube to make 4,483 measurements of the buoyant mass of another bead of known size and density in water. The width of the distribution of these measurements (120 nanograms in the right-hand distribution in Fig 1E) represents the smallest difference or change in mass that can be detected by this glass tube.

In addition to measuring the buoyant mass of samples, vibrating glass tubes can also be used to measure the absolute mass, volume, and density of a sample. By measuring the buoyant mass of the object in two fluids of different known densities, the mass, volume, and density of the object may be calculated [7]. Fig 1E shows the distribution of buoyant mass measurements for the bead in salt water (left-hand distribution) and fresh water (right-hand distribution). The bead’s average buoyant mass is 3.69 ± 0.12 *μ*g in fresh water and −4.42 ± 0.16 *μ*g in salt water. Fig 1F shows a plot of these two average buoyant mass measurements vs. the density of the fluid used in each measurement (1.00 g/mL for fresh water and 1.046 g/mL for salt water). The y-intercept of a line drawn through these two points is the absolute (*in vacuo*) mass of the bead, 180 ± 4.4 micrograms. The slope of the line is the volume of the bead, 176.3 ± 4.3 nanoliters. Finally, the x-intercept of the line is the density of the bead, 1.0210 ± 0.0005 g/mL. The measured density of the bead agrees well with the manufacturer-provided density of the bead (1.023 g/mL). Additionally, by using the measured volume of the spherical bead to calculate the diameter of the bead, we obtain a diameter of 700 *μ*m which is in good agreement with a caliper-based measurement of the diameter of the bead (710 *μ*m).

To demonstrate that vibrating tubes can be used to measure real biological samples, we used our sensors to measure the buoyant mass of single zebrafish (*D. rerio*) embryos. These millimeter-sized embryos reside in water and are too small and fragile to weigh on a conventional balance. However, using a vibrating glass tube, we weighed 474 different zebrafish embryos and obtained an average value for the buoyant mass of a zebrafish embryo, 7.59 *μ*g (S2 Fig). The width of this distribution (standard deviation = 0.56 *μ*g) provides an insight into the intrinsic variation in zebrafish embryo size and should be of value to biologists studying the mechanisms of size regulation in these organisms.

We then hypothesized that mass measurements from vibrating tubes can give insights into the health of organisms. Previous work with the microfluidic SMR mass sensor showed that single cells undergo predictable changes in mass during normal development and reproduction [5, 7, 16]. Thus, any deviation from the normal growth trajectory can be used as a signal for abnormal development or the emergence of an illness in a cell. On the other end of the size spectrum from cells, measurements of human body weight provide some of the most fundamental insights in the health and growth of a person. We hypothesize that the same relationship between mass change and organism health applies for organisms throughout the size spectrum from cells to humans.

To test this hypothesis, we used vibrating glass tubes to continuously monitor the buoyant mass of zebrafish embryos during exposure to known toxicants. Zebrafish embryos are popular vertebrate model organisms for high throughput drug discovery and screening [17, 18] and human disease modeling [19, 20]. Additionally, as marine animals, they are used extensively in assessing the toxicity of substances in aquatic environments [21]. Current methods for assessing the health of zebrafish embryo are laborious, time-consuming, require a high degree of expertise, and can be subject to human error. Even the more automated techniques, such as newly developed optical instruments with automated tracking software [22], are expensive and might only detect changes in certain developmental stages such as hatchlings that are already exhibiting physical malformations and behavioral abnormalities. Vibrating tube mass sensors could offer an economical and high-throughput alternative to these existing techniques for assessing the health of an embryo. They can also provide information on organism mass, a primary metric in toxicology that is used as a normalizing factor for dosing of toxicants [23].

Using a vibrating glass tube, we monitored the buoyant mass of single zebrafish embryos during the first 15 hours of their development starting 2 hours post-fertilization (Fig 2). A zebrafish embryo in normal water conditions (Fig 2A) shows no significant change in its mass during the monitoring period. We have observed this flat line in many other embryos in normal water conditions (S3 Fig) and therefore associate this flat line with healthy embryos. However, zebrafish embryos exposed to known toxicants demonstrate significant changes in their mass. When exposed to solutions of silver nanoparticles (Fig 2B and 2C) and copper sulfate (Fig 2D), the embryos exhibited sometimes-abrupt decreases in their buoyant mass. Additional observations of this mass decrease accompanying toxicant exposure are provided in S4 Fig. By using our sensors to look for this mass decrease in embryos exposed to suspect toxicants, this technique could be the basis for a simple, inexpensive, and high-throughput toxicity screening tool.

To validate our technique with a diverse range of biological samples, we used vibrating tube sensors to monitor the mass of individual plant seeds during imbibition and germination. Seed imbibition, or water uptake, is used in agriculture as a metric of seed health and quality [24]. Seed germination is a change in seed metabolism when imbibition starts; germination culminates with the elongation of the embryonic axis that penetrates the seed coating. The imbibition of seeds is accompanied by a rapid leakage of cellular materials (such as sugars, amino acids, and inorganic ions) and the rate of this leakage is decreased as the tissues become hydrated [25]. If water uptake by the seed is too rapid, the seed tissue might experience injury, and if the seed enters an anaerobic state, the seed might experience accumulation of toxic chemicals such as ethanol. Both situations can encourage undesirable seed dormancy and delay germination [26]. In summary, seed imbibition and germination are important phenomena in plant research, and quantitative measurements of these phenomena would be valuable in a wide range of botanical and agricultural fields.

Fig 3A–3C show seeds from three different plants: iceland poppy (*Papaver nudicaule*), oregano (*Origanum vulgare*), and foxglove (*Digitalis purpurea*). The different sizes, shapes, and surface characteristics of these seeds would complicate optical measurements of seed size. However, by continuously weighing these seeds during imbibition and sprouting, we can obtain precise measurements of seed biomass regardless of the morphology of the seed. Using a resonating glass tube mass sensor, we found that the buoyant mass of a single Iceland poppy seed remains mostly unchanged at 9 *μ*g during the first 35 hours of exposure to water and sprouting (Fig 3D). The smaller foxglove seed remains mostly unchanged at 7 *μ*g during the same time period (Fig 3E). However, the buoyant mass of the oregano seed undergoes a clear increase in buoyant mass from 6 to 9 *μ*g during the first four hours of exposure to water and germination, after which it remains constant (Fig 3F). By fitting the first four hours of the oregano seed buoyant mass to the equation of a line, we determined that the oregano seed’s buoyant mass is increasing during this period at a rate of 12 ± 0.6 nanograms per minute. Since the density of the water surrounding the oregano seed is unchanging, Eq (1) indicates that this increase in buoyant mass can be attributed to an increase in the absolute mass of the seed (due to *e.g.* biomass synthesis as the seed sprouts) or a decrease in the volume of the seed (due to *e.g.* water displacing air inside the seed as it rehydrates).

If water displacing air was responsible for the increasing buoyant mass of the oregano seed, then one would expect the density of the seed to change during imbibition. By measuring the average mass of each seed type using a population of seeds and a laboratory balance, we can use our buoyant mass measurements to estimate the density of each seed during imbibition and sprouting. This estimate assumes that the absolute mass of the seed remains unchanged during this process. Interestingly, the Iceland poppy seed (the largest of the seeds, with an average mass of 155 *μ*g) has the lowest density of the seeds: 1.06 g/mL in Fig 3G). In contrast, the foxglove seed (the smallest of the seeds, with an average mass of only 73 *μ*g) has a relatively-high density of 1.10 g/mL. Finally, the oregano seed (a medium-sized seed with an average mass of 90 *μ*g) has a density that starts low at 1.07 g/mL but increases to 1.11 g/mL during the first four hours of exposure to water, after which it remains unchanged. Measurements like these can offer fundamental insights into both the composition of these seeds and the changes occurring within the seeds as they begin to grow into a plant.

Finally, to demonstrate our technique using a biologically-relevant sample other than an organism, we used vibrating glass tubes to precisely measure the degradation rates of biodegradable materials. For many applications in medical implants, it is desirable to have materials with known degradation rates. For example, a screw for repairing a broken bone might remain intact until the bone heals and then dissolve away. However, measuring the degradation rates of slow-degrading materials is a time-consuming and labor-intensive process. Centimeter-sized samples are usually immersed in physiologically relevant fluid for weeks or months, during which time the sample is periodically removed, weighed using a conventional balance, and returned to the fluid. This process slows the development of new biomaterials and introduces the potential for human error.

By using our resonating glass tube to automatically monitor the mass of a degrading material in fluid, we can use a much smaller sample of material than would normally be required. These small (millimeter-sized) samples have a much larger surface-area-to-volume ratio than the centimeter-sized samples required by current methods. This increases the relative degradation rate of the smaller samples in fluid, making our technique capable of measuring the degradation rate of a material in hours instead of weeks or months. We used our sensors to measure the degradation rate of a sample of magnesium, a biodegradable material that has been extensively studied for potential use in medical implants [27, 28].

Fig 4 shows the buoyant masses of two millimeter-sized samples of magnesium metal as they degrade in different fluids. In neutral-pH physiologically relevant fluid (pH = 7.0; green in Fig 4) the sample of magnesium degrades relatively quickly and is effectively gone after 20 hours of continuous measurement in our sensor. The degradation products (Mg^2+^ ions) enter the fluid, where they have a negligible effect on the fluid density (as confirmed by monitoring the tube’s baseline resonance frequency over time) and therefore no effect on our measurements. We assume that the mass of magnesium *m* remaining in the sample at any given time *t* can be modeled as an exponential decay, *m* = *m*_*i*_e^−*rt*^ (where *m*_*i*_ is the starting mass of the magnesium sample at *t* = 0 and *r* is the degradation rate). By using a least-squares regression to fit the data in Fig 4, we obtained a degradation rate of 0.14 h^−1^ for the magnesium sample in pH 7.0 buffer. However, when this experiment was repeated for a similarly-sized magnesium sample in a more-alkaline buffer (pH = 8.0; red in Fig 4), the sample took nearly 120 hours to disappear completely—about six times longer than the sample in pH 7.0 buffer. Consequently, the measured degradation rate for the sample in pH 8.0 buffer, 0.021 h^−1^, is about six times lower than the rate measured in pH 7.0 buffer. This trend makes sense because magnesium reacts with acids to form magnesium ions and hydrogen gas; this reaction would be expected to accelerate the degradation of a magnesium sample at lower pH. Our measurements quantify the extent to which this pH change accelerates magnesium degradation. These results also highlight the fact that biomaterials can behave differently in different environments. By providing a fully automated and low-cost tool for measuring nanogram-scale degradation rates of biomaterials in virtually any fluid, vibrating tube sensors can accelerate the development and testing of new biomaterials for important biomedical applications.

### Discussion

The four proof-of-concept samples studied here—microbeads, embryos, seeds, and biomaterials—are representative of a wide range of samples that may be analyzed in fluid using vibrating glass tube sensors. Our technique is very versatile because all objects have fundamental physical properties like mass. Consequently, our mass sensor can be applied to problems as diverse as screening toxic substances, understanding the growth of plants, measuring the degradation of biomaterials, and many others. And unlike imaging-based measurements of size, our mass sensor is insensitive to the shape of the object. Finally, the automation, portability, and low cost of this technique make vibrating glass tubes particularly well suited for applications in the field or in resource-limited settings.

## Supporting information

S1 Fig Fluid density calibration for two different vibrating glass tube mass sensors, one obtained from a commercial fluid density meter (A; DMA 35, Anton Paar, Graz, Austria) and one homemade from glass tubing (B). While the different sizes of the tubes lead to different resonance frequencies (∼400 Hz vs. 60 Hz) for the tubes, the resonance frequency of each tube is a linear function of the density of the fluid filling the tube. The slope of each line is the fluid density calibration constant *c*_1_ for the tube (described in Eq (3)).
(EPS)

S2 Fig Distribution of buoyant mass measurements for 474 different zebrafish embryos at 2 hours post-fertilization, obtained using our vibrating glass tube sensor. The average zebrafish embryo buoyant mass is 7.59 *μ*g. The width of this distribution (standard deviation = 0.56 *μ*g) provides an insight into the intrinsic variation in zebrafish embryo size.
(EPS)

S3 Fig Additional measurements of the buoyant mass of single zebrafish embryos in water, obtained using our vibrating glass tube sensor. For most of the zebrafish embryos in water, the buoyant masses of the embryos remained unchanged during measurement (typical results shown in **A–E**). A small fraction of these embryos exhibited changes in their buoyant masses even though they were not intentionally exposed to toxicants (typical results shown in **F–H**). We attribute these results to naturally nonviable embryos that are always present in these zebrafish populations.
(EPS)

S4 Fig Additional measurements of the buoyant mass of single zebrafish embryos exposed to various known toxicants, obtained using our vibrating glass tube sensor. These toxicant-exposed embryos exhibited clear decreases in their buoyant mass during exposure (typical results shown in **A–H**).
(EPS)

S5 Fig Measuring the quality factor of a vibrating tube mass sensor. The quality factor *Q* of one of our homemade vibrating tube mass sensors was measured using the ringdown method. The glass tube (with a resonance frequency *f* = 65.2 Hz) was manually “pinged” by flicking it with a finger, and an iPhone was used to record video of the vibrating glass tube from the side (to visualize the decreasing amplitude of the vibration over time). The video was imported into the software ImageJ [12] and resliced at the tip of the tube to create this image representing the vibrational amplitude of the tube on the Y axis and video frame number on the X axis. By counting the number of frames (639) between the start of the experiment (when the vibrational amplitude *A*_0_ = 101 pixels) and the point where the amplitude has dropped to *A*_0_/*e* = 37 pixels, then dividing this frame count by the frame rate of the camera (240 frames per second), we obtain an exponential decay time *τ* = 2.66 s for this sensor. A common expression for the quality factor *Q* of a cantilever-style mass sensor with resonance frequency *f* is *Q* = *πfτ* [13]; this results in a quality factor of 545 for this vibrating tube mass sensor.
(EPS)

S6 Fig Processing typical data from our vibrating tube mass sensor. (**A**) Raw resonance frequency data from repeated measurements of a single polyethylene microbead in water (from the right-hand distribution of measurements in Fig 1E). (**B**) The raw data is filtered using a digital lowpass filter with a cutoff frequency (1.5 Hz in this case) chosen that reduces the noise in the frequency data without decreasing (“rounding off”) the measured heights of peaks in the data. After zooming in to the filtered data (**C**), peaks corresponding to individual measurements of the microbead are visible. Zooming in further on one pair of peaks (**D**) shows the ∼2 millihertz height of these peaks (corresponding to a buoyant mass of ∼3.7 *μ*g for this microbead). The peaks come in pairs because the particular vibrating tube sensor used for this measurement had a tuning-fork shape with two vibrating “U”-shaped lobes (and therefore two peaks measured per passage of the microbead through the tube).
(EPS)

S1 File Format of raw data. (PDF)

S2 File Raw data from measuring a 700 *μ*m microbead (from Fig 1E). (ZIP)

S3 File Raw data from measuring a healthy zebrafish embryo (from Fig 2A). (ZIP)

S4 File Raw data from measuring a sprouting oregano seed (from Fig 3F). (ZIP)

S5 File Raw data from measuring a sample of degrading magnesium (from Fig 4, green). (ZIP)
