# Supplementary material for: Measuring the mass, volume, and density of microgram-sized objects in fluid
Source: PLoS One. 2017 Apr 5;12(4):e0174068. doi: 10.1371/journal.pone.0174068 (PMC5381818; doi:10.1371/journal.pone.0174068)
Supplement: S1 File — (PDF) [file pone.0174068.s007.pdf]

Format of Raw Data from *Measuring the Mass, Volume, and Density of Microgram-Sized Objects in Fluid*

Shirin Mesbah Oskui, Heran C. Bhakta, Graciel Diamante,
Huinan Liu, Daniel Schlenk, and William H. Grover*

This document describes the format of the raw data obtained from our vibrating glass tube sensors. Examples of raw data from each type of experiment in the main article text are available as online *Supplementary Information*. This data includes:

- **S2_File.zip**: raw data from measuring a 700 μm microbead (from Fig 1E)
- **S3_File.zip**: raw data from measuring a healthy zebrafish embryo (Fig 2A)
- **S4_File.zip**: raw data from measuring a sprouting oregano seed (Fig 3F)
- **S5_File.zip**: raw data from measuring a sample of degrading magnesium (Fig 4, green)

Each compressed file contains several individual data files with names like `20160722T16232-freq.bin`. These names represent the time (in year, month, day, etc.) that each data file was recorded. This format allows the files to be placed in chronological order by simply sorting the files by their names.

Each individual data file contains between a few seconds and a few minutes of data. The data is saved in binary format; every 8 bytes (64 bits) of the file represents a single frequency measurement. Each measurement is the binary representation of a double-precision floating-point number that is the measured resonance frequency, using the standard big-endian ANSI/IEEE–754 encoding. For example, in the file `20160722T16232-freq.bin`, these 64 bits:

```
0100000001111101010110100110001000001100001101000000111101000101
```

represent a frequency measurement of 469.649 Hz. Many programming languages provide ways to convert this binary data into a floating-point number. For example, this Python code uses the Numpy package (`http://www.numpy.org`) to read in the whole binary file named `20160722T16232-freq.bin` and creates the corresponding array of floating-point frequency measurements:

```
import numpy
frequency_data = numpy.fromfile("20160722T16232-freq.bin",
   dtype='>f8')
print "The file contains", len(frequency_data), "measurements."
print "The first 10 measurements are", frequency_data[:10]
```

When executed, the output of this code is:

```
The file contains 281795 measurements.
The first 10 measurements are [469.65996618  469.65169456
   469.64893742  469.65996618  469.64618031  469.65720894
   469.65169456  469.64342323  469.66272346  469.64893742]
```

The data acquisition card measuring the resonance frequency of our vibrating glass tube sensor makes one frequency measurement per period of the sensor's signal. In other words, if a tube is vibrating at 469.649 Hz, then its frequency will be measured every $\frac{1}{469.649\ \mathrm{Hz}}$ seconds or every 0.002 seconds. Since the resonance frequency of the sensor changes during an experiment, the rate of frequency measurements also changes. To reconstruct the actual time at which each frequency measurement in a file was made, we take the cumulative sum of the inverse of each frequency measurement using code like this:

```
import numpy
frequency_data = numpy.fromfile("20160722T16232-freq.bin",
   dtype='>f8')
time_data = numpy.cumsum(1.0 / frequency_data)
for (time, frequency) in zip(time_data, frequency_data):
    print "Time =", time, "s \t Frequency =", frequency, "Hz"
```

When executed, the output of this code is:

```
Time = 0.0021292 s        Frequency = 469.659966184 Hz
Time = 0.0042584375 s     Frequency = 469.651694562 Hz
Time = 0.0063876875 s     Frequency = 469.648937419 Hz
Time = 0.0085168875 s     Frequency = 469.659966184 Hz
Time = 0.01064615 s       Frequency = 469.646180309 Hz
Time = 0.0127753625 s     Frequency = 469.657208945 Hz
```

Once the frequency measurements and corresponding times are loaded into arrays in this manner, the data can be filtered, searched for peaks corresponding to object mass measurements, and plotted. This was the process used to create Figs 1–4 in the main text.
